# Factors associated with transgender people mortality due to violent causes: a cross-sectional study, Brazil, 2014-2022

**DOI:** 10.1590/S2237-96222026v35e20240748.en

**Published:** 2026-03-16

**Authors:** Mayara Alves Luis, Jurema Corrêa da Mota, Camila Arantes Ferreira Brecht D’Oliveira, Cíntia Honório Vasconcelos, Ranielle de Paula Silva, Ademar Barbosa Dantas, Fernanda Lopes Regina, Cheila Marina de Lima, Naiza Bandeira de Sá, Letícia de Oliveira Cardoso

**Affiliations:** 1 Ministério da Saúde, Departamento de Análise Epidemiológica e Vigilância de Doenças Não Transmissíveis, Brasília, DF, Brazil

**Keywords:** Transgender People, Mortality, Violence, Health Information Systems, Cross-Sectional Studies, Personas Transgénero, Mortalidad, Violencia, Sistemas de Información en Salud, Estudios Transversales

## Abstract

**Objective:**

To identify factors associated with transgender people mortality due to violent causes in Brazil between 2014 and 2022.

**Methods:**

This was a cross-sectional study that matched records of reports of violence against transgender people held on the Notifiable Health Conditions Information System between 2014 and 2022 with deaths recorded on the Mortality Information System between 2014 and 2022. Frequency and proportion of reported violence were calculated, in addition to a Poisson model to estimate the prevalence ratio (PR) and the association of violence with risk factors.

**Results:**

In all, 36,452 cases of violence against transgender people were reported, of which 563 (42.5%) resulted in deaths from violent causes. Mortality due to violent causes was higher among transgender people for whom violence had been reported in the 10-14 years age group (PR 4.29; 95% confidence interval [95%CI] 2.82; 6.53) and the 15-19 age group (PR 3.82; 95%CI 2.80; 5.21), with records of self-harm (PR 1.50; 95%CI 1.28; 1.76), aggression with firearms (PR 2.00; 95%CI 1.65; 2.43) and time between reported violence and death of less than six months (PR 2.07; 95%CI 1.80; 2.39).

**Conclusion:**

The findings of this study highlighted the severity of violence against transgender people and identified individual characteristics, violence and aggression associated with mortality among transgender people in Brazil.

## Resumen

**Objetivo:**

Identificar los factores asociados a la mortalidad de personas transgénero por causas violentas en Brasil entre 2014 y 2022.

**Métodos:**

Estudio transversal que emparejó los registros de denuncias de violencia contra personas transgénero en el Sistema de Información de Enfermedades de Notificación Obligatoria entre 2014 y 2022, con las muertes registradas en el Sistema de Información de Mortalidad entre 2014 y 2022. Se calculó la frecuencia y la proporción de denuncias, además de un modelo de Poisson para estimar la razón de prevalencia (RP) y la asociación de la violencia con factores de riesgo.

**Resultados:**

Se notificaron 36.452 casos de violencia contra personas transgénero, de los cuales 563 (42,5%) fueron muertes por causas violentas. La mortalidad por causas violentas fue mayor entre las personas transgénero con registro de violencia en el grupo de edad de 10 a 14 años (RP 4,29; intervalo de confianza del 95% [IC95%] 2,82; 6,53) y 15 a 19 años (RP 3,82; IC95% 2,80; 5,21), con registros de autolesiones (RP 1,50; IC95% 1,28; 1,76), medio de agresión por arma de fuego (RP 2,00; IC95% 1,65; 2,43) y tiempo entre registro de violencia y muerte menor a seis meses (RP 2,07; IC95% 1,80; 2,39).

**Conclusión:**

Los hallazgos de este estudio destacaron la gravedad de la violencia contra las personas transgénero e identificaron características individuales, violencia y agresión asociadas con la mortalidad en la población transgénero en Brasil.

**Ethical aspects**

This research respected ethical principles, having obtained the following approval data:

Research ethics committee: Brazilian Ministry of Health

Opinion number: 046,934,2272023

Approval date: 14/7/2024

Certificate of submission for ethical appraisal: 77188124.7.0000.0008

Informed consent form: Not applicable. The research used secondary data from Ministry of Health information systems.

## Introduction 

With effect from 2007, the National Health Council has recognized sexual orientation and gender identity as social determinants of health, highlighting the need for specific health policies for this population. Sexual orientation refers to the emotional, romantic or sexual attraction a person feels for others, and can be heterosexual, homosexual, bisexual or other. Gender identity refers to a person’s internal perception of themself as male, female, both or neither, and may or may not correspond to the sex assigned to them at birth. Transvestite is a gender identity of people who express characteristics socially associated with femininity, opposing their gender assigned at birth (1).

In 2011, the Ministry of Health published the National Policy for Comprehensive Health for Lesbian, Gay, Bisexual, Transvestite and Transgender People, a landmark measure aimed at ensuring respect and attention for the needs of this population within the Brazilian Unified Health System. This policy represented an important milestone in recognizing the demands of the community comprised of lesbian, gay, bisexual, transgender, queer, intersex, asexual people, and people of other sexual orientations and gender identities (LGBTQIA+) (2).

Violence against this community poses a significant challenge, with direct consequences for individual and collective health. The LGBTQIA+ health policy seeks to improve information about the health of this population, including surveillance of violence against them (3). In 2014, significant progress was made in this regard, with the inclusion of fields on sexual orientation, gender identity and chosen name on the violence reporting form of the Notifiable Health Conditions Information System (*Sistema de Informação de Agravos de Notificação*, SINAN), in addition to expanding the field for reasons for violence, including LGBTphobia (4).

Despite government efforts, violence against the LGBTQIA+ community continues to be a phenomenon of great magnitude. Between 2008 and 2018, nearly 3,000 homicides of transgender people were recorded in 72 countries (5), while in Brazil, between 2017 and 2022, there were 1,057 homicides of transgender, transvestite and non-binary people (6). In 2023, the human rights violation hotline, Disque 100, received 2,536 reports, a 348.8% increase compared to the 565 reports recorded in the same period in 2022 (7).

The National Transvestite and Transsexual Association (*Associação Nacional de Travestis e Transexuais*) plays an important role in publicising the number of victims in its annual dossiers. However, limited official data contributes to the invisibility of violence against these individuals (6). The lack of accurate and timely information on morbidity and mortality arising from violence against transgender people is due, among other factors, to difficulties in reporting these cases and interoperability between health information systems.

The objective of this study was to identify factors associated with transgender people mortality due to violent causes in Brazil between 2014 and 2022, by matching the Mortality Information System (*Sistema de Informações sobre Mortalidade*, SIM) and the SINAN system databases.

## Methods 

### Design 

This is an observational, analytical and cross-sectional study on the transgender population using secondary data on reports of interpersonal and self-inflicted violence, as well as mortality, in Brazil, from 2014 to 2022.

### Setting 

The time frame for this study began in 2014, the year in which interpersonal and self-inflicted violence reports began to include information on the sexual orientation and gender identity of the person assisted, thus enabling identification of violence among the transgender population (3). In contrast, death certificates, the standard document for recording information on the SIM system, continue to have no information on sexual orientation and gender identity that could identify causes of death in this population. This study matched the violence and mortality databases for the trans population and analyzed the causes of death.

### Participants 

Using data from SIM and SINAN, the study analyzed individuals over the age of 10 and residing in Brazil. With regard to SIM data, deaths from 2014 to 2022 were included, regardless of the underlying or secondary cause of death or sex at birth. As for SINAN data, individuals who self-identified as transvestites, transgender women or transgender men and who had a record of interpersonal or self-inflicted violence between 2014 and 2022 were included.

### Variables 

The main outcome was the death of transgender individuals reported on SINAN and on SIM, classified as external or non-external causes. Exposure variables included: gender identity; age group; race/skin color; schooling; marital status; presence of disability/disorder; location of occurrence; time of day; recurrence of the event; relationship between aggressor and victim; type of violence and means of aggression; time between the first report of violence on SINAN and date of death on SIM.

### Data sources and measurement 

Reports of interpersonal and self-inflicted violence were obtained from SINAN, from 2014-2022, while death records were obtained from SIM, in the same period, for Brazil as a whole.

Deaths were classified according to the chapters and causes contained in the tenth revision of the International Classification of Diseases (ICD-10). The causes were grouped into violent – ​​under the codes: X85-Y09, Y35, Y87.1 (homicide); X60-X84, Y87.0 (suicide); and Y10-Y34 (undetermined causes) – and non-violent, which included the remaining ICD-10 codes.

The variables used in relation to reports of violence were: gender identity (transgender woman/transvestite, transgender man); reporting date; age group (10-14, 15-19, 20-39, 40-59, 60 years and older); race/skin color (White, Black, Brown [Brazilian mixed race], Asian, Indigenous); schooling (illiterate, up to eight years of schooling, more than eight years of schooling); marital status (had a partner, did not have a partner); disability/disorder (yes, no); place where the violence occurred (residence, public street, other); time of day when the violence occurred (morning, afternoon, evening, early hours); whether violence had occurred previously (yes, no); aggressor/victim relationship (partner, family member, acquaintance, stranger, the person themself, others); type of violence (self-inflicted, physical, psychological, physical and psychological, sexual, torture, financial, other, unknown); and means of aggression (bodily force, threat, threat and bodily force, firearm, sharp object, hanging, blunt object, other, unknown). Because it is possible to record more than one type of violence and means of aggression for each report, these variables were classified so as to remain in mutually exclusive categories (8).

The time between the first report of violence and death was classified as up to six months or more than six months, using the difference between the date of the first report of violence on SINAN and the date of death on SIM. Reports of violence and mortality records were used for matching, in addition to the following variables: victim’s name, mother’s name, date of birth, and municipality of residence.

### Bias control

Mortality was classified as being due to violent and non-violent causes following ICD-10 recommendations. In order to minimize potential classification bias in mortality records for vulnerable populations, undetermined causes were included as part of violent causes, as the literature indicates that deaths classified as being due to undetermined causes migrate to suicide or homicide after matched analysis (9) and death investigation (10).

### Study size

Of the 3,034,939 reports of violence recorded on SINAN from 2014 to 2022, 37,533 were included, related to victims who self-identified as transgender people, according to the eligibility criteria. After restructuring the SINAN database in a longitudinal format, 36,452 cases of interpersonal or self-inflicted violence were considered for data matching and analysis. Matching with SIM retrieved 1,325 deaths from violent and non-violent causes among the 16,606,876 deaths recorded in the period 2014 to 2022.

### Statistical methods

The simple and relative frequencies of covariates were compared according to death from violent causes using the chi-square test and a 5% significance level. Causes of death, according to ICD-10 chapters, were analyzed using relative frequencies. Associations between covariates and death were measured by prevalence ratios (PR) with their respective 95% confidence intervals (95%CI) using Poisson regression. Goodness-of-fit was assessed using pseudo R^2^, likelihood ratio and the corrected Akaike information criterion. All analyses were conducted using the blorr package (11) run on the R program.

### Data access and cleaning methods 

Reports of interpersonal and self-inflicted violence and death records with identification of names were obtained by means of a request made to the Health and Environmental Surveillance Secretariat, with the study authors signing the responsibility and confidentiality record regarding the transfer of the databases. Both the SINAN database and the SIM database periodically undergo consistency and missing data analysis.

### Data matching

SINAN allows more than one report for the same individual, which is why we transposed the reports longitudinally, thus enabling observation of the individual over time. In other words, the database was no longer composed of reports but rather of individual records. In order to perform report transposition, the variables considered in data matching were used: victim’s name, date of birth, mother’s name and municipality of residence.

After longitudinal transposition, linkage between SIM and SINAN used the aforementioned variable fields and was conducted in four stages.

In the first stage, in the longitudinal SINAN and SIM databases, the names of the individual and their mother were standardized, removing special characters, double spaces, accents, connectors, prepositions and blank spaces, as well as fields related to date of birth. A unique numerical sequence index was created for these databases, and they were reduced into one copy, containing only the fields necessary for matching.

Taking these reduced databases, the second stage was to separate the names into first, last, and middle names of both the victim and the victim’s mother. Records without the individual’s name were excluded from both reduced databases.

The third stage was deterministic linkage between the reduced SIM and SINAN databases. A unique key was created consisting of the complete fields: victim’s name, date of birth, sex, mother’s name and municipality of residence. This key was used to match the databases, assuming 100% similarity, using the left_join function in the R program. In other words, death information based on the unique key matching was added to the reduced SINAN database.

The fourth step involved linkage of records (not previously matched) by probabilistic matching, using: first name, middle name and last name of the individual and their mother, sex, date of birth and municipality of residence. The probabilistic matching was performed using the R program RecordLinkage package (12), which enabled the name fields to be transformed into phonetic code, by using SoundexBR (13).

During matching, potential pairs were weighted, which would enable a cutoff point to be established in order to verify record similarity. However, as the study population was specific, we decided not to establish a cutoff point. The RecordLinkage package enabled potential matches to be exported as a CSV file. Using this file, the records were manually inspected to ensure identification and validation of true matches.

After the fourth stage, the pairs identified via probabilistic matching were integrated into the reduced databases using unique indexers, which already contained the deterministically identified pairs. Finally, using the left_join function, this database was integrated into the larger SINAN database.

## Results 

Between 2014 and 2022, 36,452 cases of interpersonal violence or self-harm against transgender people were reported; 1,325 were associated with deaths (3.6% of total reports) and, of these, 563 (42.5%) were due to violence (homicide or suicide). There was an increase in reports over the years, from 1.3% in 2014 to 16.2% in 2022 (Table 1).

**Table 1:** Frequency and proportion of reports of violence and deaths of trans people, retrieved through deterministic and probabilistic matching processes, according to year reported and by violent and non-violent causes. Brazil, 2014-2022 (n=36,452)

Year	Reports	Deaths		
		Total	Violent causes	Other causes
	n (%)	n (%)	n (%)	n (%)
2014	491 (1.3)	20 (4.1)	19 (95.0)	1 (5.0)
2015	3,137 (8.6)	63 (2.0)	47 (74.6)	16 (25.4)
2016	4,285 (11.8)	99 (2.3)	54 (54.5)	45 (45.5)
2017	4,067 (11.2)	134 (3.3)	66 (49.3)	68 (50.7)
2018	4,654 (12.8)	135 (2.9)	52 (38.5)	83 (61.5)
2019	4,802 (13.2)	174 (3.6)	81 (46.6)	93 (53.4)
2020	4,269 (11.7)	205 (4.8)	66 (32.2)	139 (67.8)
2021	4,825 (13.2)	240 (5.0)	82 (34.2)	158 (65.8)
2022	5,922 (16.2)	255 (4.0)	96 (37.6)	159 (62.4)
Total	36,452 (100.0)	1,325 (3.6)	563 (42.5)	762 (57.5)

**Figure 1:**
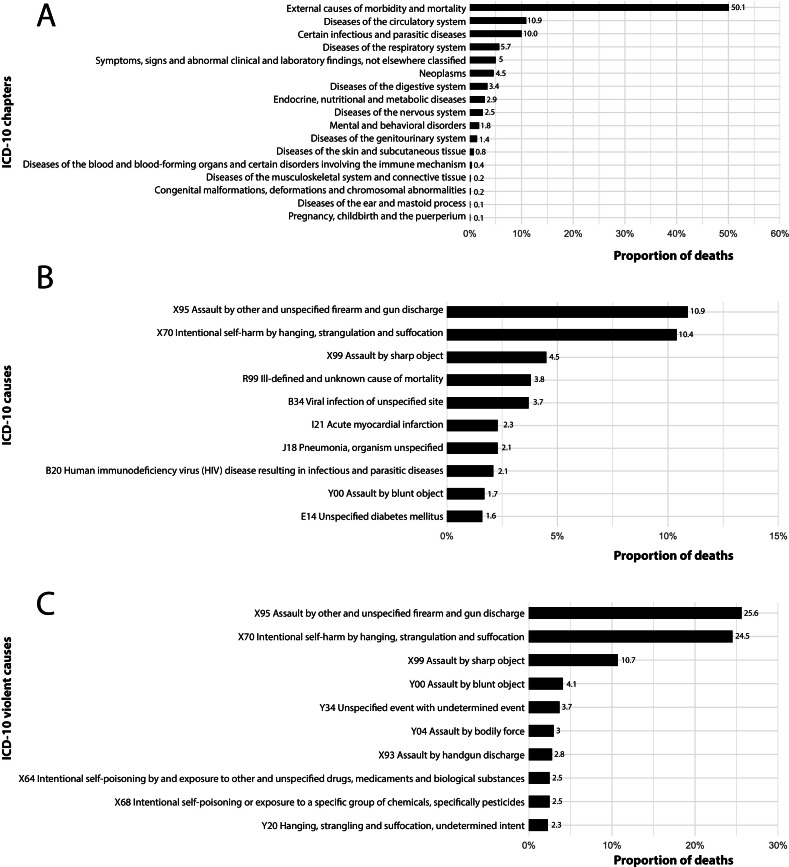
Proportion of the underlying causes of death of transgender people who suffered violence: (A) Proportion of deaths as per tenth revision of the International Classification of Diseases (ICD-10) chapters; (B) ICD-10 causes; (C) ICD-10 external causes. Brazil, 2014-2022 (n=1,325)

Grouped together according to ICD-10 chapter, external causes accounted for 50.1% of all deaths; circulatory system diseases, 10.9%; and infectious and parasitic diseases, 10.0% (Figure 1). Considering the ten most common causes of death, the most prominent were assaults with firearms (10.9%) and suicide by hanging, strangulation or suffocation (10.4%). Homicides with firearms and suicide by hanging, strangulation or suffocation accounted for 50.0% of all violent deaths (Figure 1).

The analysis of the notifications recorded in Sinan of transgender people who died from violent causes showed that most of the reported cases were among trans women and travestis (73.5%), individuals aged 20 to 39 years (54.2%), those identified as Brown/Pardo (50.6%), and those with up to eight years of schooling (45.3%). It was also observed that most of these individuals did not have a partner (67.6%) and had no disability or disorder recorded in the notification (79.8%) (Table 2).

**Table 2:** Frequency and proportion of characteristics of transgender people for whom violence was reported, according to death from violent (n=563) and non-violent (n=762) causes. Brazil, 2014-2022 (n=1,325)

Variables	Death from violent causes		p-value
	Yes^a^	No^b^	
	n (%)	n (%)	
**Gender identity**			0.006
Transgender woman and transvestite	414 (73.5)	609 (79.9)	
Transgender man	149 (26.5)	153 (20.1)	
**Age group** (years)			<0.001
10-14	14 (2.5)	8 (1.0)	
15-19	88 (15.6)	34 (4.5)	
20-39	305 (54.2)	210 (27.6)	
40-59	115 (20.4)	183 (24.0)	
60+	41 (7.3)	327 (42.9)	
**Race/skin color**			0.007
White	191 (35.4)	294 (41.2)	
Black	55 (10.2)	75 (10.5)	
Asian	3 (0.6)	6 (0.8)	
Brown	273 (50.6)	333 (46.6)	
Indigenous	18 (3.3)	6 (0.8)	
Schooling			<0.001
Elementary education	222 (45.3)	333 (49.0)	
High school education	82 (16.7)	64 (9.4)	
Higher education	22 (4.5)	15 (2.2)	
Unknown	164 (33.5)	268 (39.4)	
**Marital status**			0.116
Had a partner	165 (32.4)	241 (36.9)	
Did not have a partner	344 (67.6)	413 (63.1)	
**Disability or disorder**			0.458
Yes	102 (20.2)	154 (22.0)	
No	403 (79.8)	547 (78)	
**Place of occurrence**			<0.001
Residence	299 (55.8)	521 (71.6)	
Public street	165 (30.8)	133 (18.3)	
Other	72 (13.4)	74 (10.2)	
**Time of day**			0.003
Morning	75 (19.8)	90 (21.0)	
Afternoon	76 (20.1)	129 (30.1)	
Night	140 (37.0)	142 (33.1)	
Early hours	87 (23.0)	68 (15.9)	
**Occurred previously**?			<0.001
Yes	158 (34.3)	293 (45.5)	
No	303 (65.7)	351 (54.5)	
**Self-inflicted injury**			<0.001
Yes	243 (44.8)	148 (20.9)	
No	299 (55.2)	561 (79.1)	
**Type of violence**			<0.001
Physical	336 (61.4)	336 (45)	
Psychological	8 (1.5)	19 (2.5)	
Physical+psychological	36 (6.6)	83 (11.1)	
Sexual	20 (3.7)	28 (3.8)	
Torture	22 (4.0)	24 (3.2)	
Financial	3 (0.5)	20 (2.7)	
Other	122 (22.3)	236 (31.6)	
**Type of aggression**			<0.001
Bodily force	96 (17.6)	233 (34.1)	
Threat	3 (0.5)	15 (2.2)	
Threat+bodily force	10 (1.8)	31 (4.5)	
Firearm	112 (20.5)	13 (1.9)	
Sharp object	90 (16.5)	92 (13.5)	
Hanging	98 (17.9)	26 (3.8)	
Blunt object	23 (4.2)	37 (5.4)	
Other	115 (21.0)	237 (34.6)	
**Aggressor/victim relationship**			<0.001
Partner	56 (11.2)	113 (16.2)	
Acquaintance	64 (12.8)	69 (9.9)	
Family member	22 (4.4)	179 (25.7)	
Stranger	116 (23.2)	105 (15.1)	
Person themself	211 (42.1)	142 (20.4)	
Other	32 (6.4)	88 (12.6)	
**Time between report and death** (months)			<0.001
Up to 6	377 (67.0)	197 (25.9)	
More than 6	186 (33.0)	565 (74.1)	

^a^563 (42,5%); ^b^762 (57,5%).

Regarding the characteristics of the reported occurrences, 55.8% of the cases took place in the victim’s residence, 37.0% occurred at night, and 65.7% were first-time events with no previous notifications. Of all cases, 44.8% involved self-inflicted violence and 55.2% involved interpersonal violence. Among interpersonal violence cases, physical violence was the most frequent, accounting for 61.4% of notifications.

The main means of aggression used in these episodes were firearms (20.5%), hanging (17.9%), physical force (17.6%), and sharp objects (16.5%). Regarding the relationship with the aggressor, 23.2% of cases were attributed to strangers, 12.8% to acquaintances, and 11.2% to intimate partners.

It is important to note that these characteristics refer exclusively to previously reported episodes of violence and do not necessarily correspond to the aggression that resulted in the death.

The results on deaths indicated that certain characteristics increased the likelihood of dying from violent causes among trans people who had, at some point prior, a recorded notification of violence in the Sinan system.

The findings showed that the prevalence of violent death was higher among younger trans individuals: ages 10-14 (PR 4.29; 95%CI 2.82; 6.53), 15-19 (PR 3.82; 95%CI 2.80; 5.21), 20-39 (PR 3.37; 95%CI 2.52; 4.50), and 40-59 (PR 2.62; 95%CI 1.94; 3.54), compared with those aged 60 years or older.

Among the characteristics of previous notifications, the risk of violent death was higher in individuals with a record of self-inflicted injury (PR 1.50; 95%CI 1.28; 1.76) and in those who had suffered aggression involving firearms (PR 2.00; 95%CI 1.65; 2.43), hanging (PR 1.59; 95%CI 1.36; 1.88), blunt objects (PR 1.56; 95%CI 1.10; 2.21), or sharp objects (PR 1.48; 95%CI 1.20; 1.83).

Additionally, people whose first notification occurred less than six months before death had a higher prevalence of violent mortality (PR 2.07; 95%CI 1.80; 2.39), indicating increased vulnerability in the period immediately following the notification.

## Discussion 

The results indicated a progressive increase in reports of violence against trans people. Among the cases recorded in Sinan that later resulted in death in the SIM system, there was a higher frequency of violent deaths among individuals aged 10 to 14, those with a prior notification of self-inflicted injuries, and those who had been victims of assaults involving firearms, sharp objects, and hanging.

Table 3

**Table 3:** Unadjusted and adjusted prevalence rations (PR) and 95% confidence intervals (95%CI) for deaths from violent causes among transgender persons for whom violence was reported, by study variables. Brazil, 2014- 2022 (n=1,325)

Variables	Unadjusted PR (95%CI)	p-value	Adjusted PR (95%CI	p-value
**Gender identity**				
Transgender woman and transvestite	1,22 (1,06; 1,40)	0,038		
Transgender man	1,00			
**Age group (years)**				
10-14	5,71 (3,72; 8,76)	<0,001	4,29 (2,82; 6,53)	<0,001
15-19	6,47 (4,75; 8,82)	<0,001	3,82 (2,80; 5,21)	<0,001
20-39	5,31 (3,95; 7,16)	<0,001	3,37 (2,52; 4,50)	<0,001
40-59	3,46 (2,51; 4,78)	<0,001	2,62 (1,94; 3,54)	<0,001
60+	1,00		1,00	
**Marital status**				
Had a partner	1,00			
Did not have a partner	1,12 (0,97; 1,29)	0,238		
**Place of occurrence**				
Residence	0,74 (0,61; 0,89)	0,021		
Public street	1,12 (0,92; 1,36)	0,412		
Other	1,00			
**Self-inflicted injury**				
Yes	1,79 (1,58; 2,01)	<0,001	1,50 (1,28; 1,76)	<0,001
No	1,00		1,00	
**Type of violence**				
Physical	1,47 (1,25; 1,73)	<0,001		
Psychological	0,87 (0,65; 1,21)	0,701		
Physical+psychological	0,89 (0,65; 1,21)	0,530		
Sexual	1,22 (0,85; 1,76)	0,404		
Torture	1,40 (1,00; 1,96)	0,143		
Financial	0,38 (0,13; 1,11)	0,100		
Other	1,00			
**Type of aggression**				
Bodily force	0,89 (0,71; 1,12)	0,413	1,28 (1,01; 1,62)	0,133
Threat	0,51 (0,18; 1,45)	0,249	0,67 (0,24; 1,87)	0,573
Threat+bodily force	0,75 (0,43; 1,31)	0,375	1,23 (0,74; 2,06)	0,541
Firearm	2,74 (2,33; 3,22)	<0,001	2,00 (1,65; 2,43)	<0,001
Sharp object	1,51 (1,23; 1,87)	0,003	1,48 (1,20; 1,83)	0,009
Hanging	2,42 (2,03; 2,88)	<0,001	1,59 (1,36; 1,88)	<0,001
Blunt object	1,17 (0,82; 1,67)	0,484	1,56 (1,10; 2,21)	0,077
Other	1,00		1,00	
**Aggressor/victim relationship**				
Partner	1,24 (0,86; 1,79)	0,326		
Acquaintance	1,80 (1,28; 2,55)	0,006		
Family member	0,41 (0,25; 0,67)	0,001		
Stranger	1,97 (1,43; 2,72)	<0,001		
Person themself	2,24 (1,64; 3,05)	<0,001		
Other	1,00			
**Time between report and death (months)**				
Up to 6	2,65 (2,31; 3,04)	<0,001	2,07 (1,80; 2,39)	<0,001
More than 6	1,00		1,00	

Among the study’s limitations were those related to the use of secondary data, such as case underreporting, data incompleteness and inconsistency, which can compromise the quality of the analyses and lead to bias.

The findings of this study were limited to the mortality of transgender people with reported violence in Brazil, therefore, they did not reveal the actual violence suffered by transgender people. This was the first study to use data from SINAN and SIM to explore the topic of violence against transgender people in Brazil. The novelty of these findings illuminated not only the factors associated with transgender mortality but also the need for further studies on the topic, especially regarding gender, life expectancy and the relationship between suicide, gender and age group.

The inclusion of the gender identity field on the violence reporting form between 2014 and 2015 resulted in an increase in SINAN records over the years. The results suggest that this increase may be due to training for professionals who report these events, which results in greater sensitivity in identifying and reporting violence that occurs in this population, in addition to better field completion. The expanded coverage of reporting municipalities may also have contributed to the increase in the number of cases of violence against transgender men and women and transvestites.

Although this study did not identify statistical association between gender identity and death from violent causes, most cases occurred among transgender women and transvestites. Homicides occur more frequently among transgender women compared to transgender men (6,14,15). This discrepancy revealed the intersection of violence such as transphobia, misogyny and racism, which increased their vulnerability (6,14,16).

Prevalence of death from violent causes was higher in all age groups when compared to people aged 60 and over, although decreasing as age increased. On average, life expectancy of transgender people has been found to be seven years less than that of non-trans people (18). Therefore, the higher incidence of death by homicide or suicide among young people may indicate vulnerabilities related to lack of support, discrimination, financial insecurity, body dissatisfaction and emotional issues (17).

Physical violence was present in most of the notifications referring to trans people who subsequently died. Following verbal aggression, such as insults and swearing, physical violence constituted the main type of harm registered, corresponding to 62% of the cases (19).

The most frequent causes of death among trans people were assault by firearm, suicide by hanging, and assault by sharp/piercing objects. The analysis of associated factors indicated that violent deaths were more recurrent among those who had records of hanging, assault by blunt and sharp/piercing objects, and especially assaults involving firearms in their notifications. This pattern contrasted with that of the general population, whose main causes of death were related to cardiovascular disease and neoplasms (20).

Transgender individuals, especially those under 30 years of age, were the main targets of murder in this community, in addition to suffering more injuries than necessary to result in death, often perpetrated through sharp weapons, beatings, asphyxiation and firearms (21). Victims of aggression by these means should be carefully monitored by professionals responsible for providing care and referral to the protection network, as the use of these methods may indicate greater aggression on the part of the aggressor, which increases the risk of recurring violence and subsequent death (8).

Firearm ownership has been associated with an increase in lethal violence in Brazil (22). Law No. 13,880/2019, by making ownership more flexible, has contributed to the growth in the number of registered firearm collectors, shooters and hunters. Therefore, it is essential to implement policies that restrict access to weapons as a strategy for combating lethal violence.

Another study showed that 29% of trans people had already attempted suicide during their lifetime, which is higher when compared to studies conducted in the general population (23). In Brazil, among 278 transgender people participating in a study, this prevalence rate was 43.1%. The high incidence of mental disorders in this population, especially depression, stood out (25).

Transphobia negatively impacts individuals’ physical and mental health. This reality can manifest itself through institutional violence, as well as family conflicts, often resulting in isolation from social environments and loss of housing or shelter (26). Over the course of a lifetime, exposure to violence due to prejudice, discrimination and stigmatization can negatively impact the mental health of victims, contributing to greater risk of suicidal behavior and interpersonal violence (27,28).

This study identified higher prevalence of deaths from violent causes in the six months after the first report of violence and highlighted the need for rapid responses to protect the physical integrity of victims, prevent the worsening of violence and deaths and ensure the safety of transgender people in situations of violence.

Despite the lack of official data that allows for the precise dimensioning of the trans population in the country, the findings of this study highlighted the severity of mortality from violent causes in this group, as well as the fragility of information systems in capturing, qualifying, and integrating essential data for the health surveillance of this population. The findings reinforce the urgency of improving information systems to allow for a greater understanding of the contexts of violence that affect the trans population. Furthermore, they evidence the need for inclusive public policies, especially aimed at younger individuals, that promote visibility, protection, and guaranteed rights, focusing on increasing the life expectancy and quality of life of trans people.

The high prevalence of mortality from violent causes among individuals with records of self-harm, combined with the short interval between reported violence and death, highlights the importance of active surveillance for timely intervention. It is crucial that health services are prepared to offer qualified support and comprehensive assistance, ensuring timely and effective responses to identified cases. Therefore, it is essential that surveillance and care professionals are trained to recognize situations of greater vulnerability and make appropriate referrals to specialized services and the protection network.

## Data Availability

Given that the data are sensitive and identifiable, the databases used in this research have not been made available in public repositories.
