# Supplementary material for: Factors associated with transgender people mortality due to violent causes: a cross-sectional study, Brazil, 2014-2022
Source: Epidemiol Serv Saude. 2026 Mar 16;35:e20240748. doi: 10.1590/S2237-96222026v35e20240748.en (PMC12995283; doi:10.1590/S2237-96222026v35e20240748.en)
Supplement: Translation

# Translation: Fatores associados à mortalidade de pessoas trans por causas violentas: estudo transversal, Brasil, 2014-2022

**DOI:** 10.1590/S2237-96222026v35e20240748.pt

**Authors:** Mayara Alves Luis, Jurema Corrêa da Mota, Camila Arantes Ferreira Brecht D’Oliveira, Cíntia Honório Vasconcelos, Ranielle de Paula Silva, Ademar Barbosa Dantas, Fernanda Lopes Regina, Cheila Marina de Lima, Naiza Bandeira de Sá, Letícia de Oliveira Cardoso

**Affiliations:** 1 Ministério da Saúde, Departamento de Análise Epidemiológica e Vigilância de Doenças Não Transmissíveis, Brasília, DF, Brasil

**Keywords:** Pessoas Transgênero, Mortalidade, Violência, Sistemas de Informação em Saúde, Estudo Transversais

## Abstract

**Objetivo:**

Identificar fatores associados à mortalidade de pessoas trans por causas violentas no Brasil entre 2014 e 2022.

**Métodos:**

Estudo transversal, no qual foi realizado pareamento dos registros de notificações de violência contra pessoas trans no Sistema de Informação de Agravos de Notificação entre 2014 e 2022, bem como de óbitos registrados no Sistema de Informações sobre Mortalidade entre 2014 e 2022. Foram calculadas frequência e proporção de notificações, além de modelo de Poisson, para estimar a razão de prevalência (RP) e a associação da violência com fatores de risco.

**Resultados:**

Foram notificados 36.452 casos de violência contra pessoas trans, dos quais 563 (42,5%) se constituíram de óbitos por causa violenta. A mortalidade por causas violentas foi maior entre as pessoas trans com notificação de violência na faixa etária de 10-14 anos (RP 4,29; intervalo de confiança de 95% [IC95%] 2,82; 6,53) e de 15-19 anos (RP 3,82; IC95% 2,80; 5,21), com registro de lesão autoprovocada (RP 1,50; IC95% 1,28; 1,76), meio de agressão por arma de fogo (RP 2,00; IC95% 1,65; 2,43) e tempo entre notificação e óbito menor que seis meses (RP 2,07; IC95% 1,80; 2,39).

**Conclusão:**

Os achados deste estudo evidenciaram a gravidade da violência contra pessoas trans e identificaram características individuais, da violência e da agressão associadas à mortalidade da população trans no Brasil.

**Aspectos éticos**

Esta pesquisa respeitou os princípios éticos, obtendo os seguintes dados de aprovação:

Comitê de ética em pesquisa: Ministério da Saúde

Número do parecer: 6.934.227

Data de aprovação: 14/7/2024

Certificado de apresentação de apreciação ética: 77188124.7.0000.0008

Registro do consentimento livre e esclarecido: Não se aplica. A pesquisa utilizou dados secundários dos sistemas de informação do Ministério da Saúde.

## Introdução 

Desde 2007, o Conselho Nacional de Saúde reconhece a orientação sexual e a identidade de gênero como determinantes sociais da saúde, o que evidencia a necessidade de políticas de saúde específicas para essa população. A orientação sexual refere-se à atração emocional, romântica ou sexual que uma pessoa sente por outras, podendo ser heterossexual, homossexual, bissexual, entre outras. Já a identidade de gênero diz respeito à percepção interna que uma pessoa tem de si mesma como homem, mulher, ambos ou nenhum, podendo ou não corresponder ao sexo designado ao nascer, enquanto travesti é uma identidade de gênero de pessoas que expressam características socialmente associadas ao feminino, oposta ao gênero de nascimento (1). 

Em 2011, o Ministério da Saúde publicou a Política Nacional de Saúde Integral de Lésbicas, Gays, Bissexuais, Travestis e Transexuais, uma medida histórica que visava garantir o respeito e a atenção às necessidades dessa população no âmbito do Sistema Único de Saúde. Essa política representou um marco importante no reconhecimento das demandas da comunidade lésbicas, gays, bissexuais, transexuais, queer, intersexuais, assexuais e demais orientações sexuais e identidades de gênero (LGBTQIA+) (2).

A violência contra essa comunidade representa um desafio significativo, com consequências diretas para a saúde individual e coletiva. A política de saúde LGBTQIA+ busca melhorar a informação sobre a saúde dessa população, incluindo a vigilância das violências contra ela (3). Em 2014, um importante avanço ocorreu nesse sentido, com a inclusão de campos sobre orientação sexual, identidade de gênero e nome social na ficha de notificação de violência do Sistema de Informação de Agravos de Notificação (Sinan), além de haver a ampliação do campo para motivos de violência, incluindo a LGBTfobia (4).

Apesar dos esforços governamentais, a violência contra a comunidade LGBTQIA+ permanece como um fenômeno de alta magnitude. Entre 2008 e 2018, foram registrados quase 3 mil homicídios de pessoas trans em 72 países (5), enquanto, no Brasil, entre 2017 e 2022, ocorreram 1.057 homicídios de pessoas trans, travestis e não binárias (6). Em 2023, o Disque 100 recebeu 2.536 denúncias, um aumento de 348,8% em comparação às 565 denúncias registradas no mesmo período em 2022 (7).

A Associação Nacional de Travestis e Transexuais desempenha importante papel na contagem de vítimas por meio de relatórios anuais, contudo a limitação na produção de dados oficiais contribui para a invisibilidade das violências contra essas pessoas (6). A escassez de informações precisas e oportunas sobre morbimortalidade por violências contra as pessoas trans se deve, entre outros fatores, pelas dificuldades nas notificações desses casos e na interoperabilidade entre os sistemas de informação em saúde.

O objetivo deste estudo foi identificar fatores associados à mortalidade das pessoas trans por causas violentas no Brasil entre 2014 e 2022, a partir do pareamento das bases de dados do Sistema de Informações sobre Mortalidade (SIM) e do Sinan.

## Métodos 

### Delineamento do estudo

Trata-se de estudo observacional, analítico e transversal sobre a população trans com dados secundários de notificações de violência interpessoal e autoprovocada, além de mortalidade, no Brasil, de 2014 a 2022.

### Contexto 

O recorte temporal deste estudo iniciou-se em 2014, ano em que as notificações de violência interpessoal e autoprovocada passaram a incluir informações sobre orientação sexual e identidade de gênero da pessoa atendida, permitindo a identificação da violência na população trans (3). Em contrapartida, a declaração de óbito, documento-padrão para registro de informações no SIM, permanece sem informações de orientação sexual e identidade de gênero que possam identificar causas de óbitos nessa população. O estudo pareou as bases de dados de violência e de mortalidade para a população trans e analisou as causas de óbito.

### Participantes 

A partir de dados do SIM e do Sinan, foram considerados indivíduos com idade superior a 10 anos e residentes no Brasil. Do SIM, foram incluídos óbitos de 2014 a 2022, independentemente da causa básica ou secundária de morte e do sexo ao nascimento. Do Sinan, foram incluídos indivíduos autodeclarados travestis, mulheres transexuais ou homens transexuais e que tiveram registro de violência interpessoal ou autoprovocada entre 2014 e 2022.

### Variáveis 

O desfecho principal foi o óbito de pessoas trans notificadas no Sinan e declarado no SIM, classificado como causas externas e não externas. As variáveis de exposição incluíram: identidade de gênero; faixa etária; raça/cor da pele; escolaridade; situação conjugal; presença de deficiência/transtorno; local da ocorrência; turno do dia; reincidência do evento; vínculo entre agressor e vítima; tipo de violência e meios de agressão; tempo entre a primeira notificação no Sinan e o óbito no SIM.

### Fontes de dados e mensuração

As notificações de violência interpessoal e autoprovocada foram obtidas do Sinan, de 2014-2022, e os registros de óbitos do SIM, no mesmo período, no Brasil.

Os óbitos foram classificados em capítulos e causas, segundo a décima revisão da Classificação Internacional de Doenças (CID-10). As causas foram agrupadas em violentas – sob os códigos: X85-Y09, Y35, Y87.1 (homicídios); X60-X84, Y87.0 (suicídios); e Y10-Y34 (causas indeterminadas) – e em não violentas, que abrangeu os demais códigos da CID-10. 

As variáveis utilizadas, em relação às notificações de violência, foram: identidade de gênero (mulher trans/travesti, homem trans); data de notificação; faixa etária (10-14, 15-19, 20-39, 40-59, 60 anos e mais); raça/cor da pele (branca, preta, parda, amarela, indígena); escolaridade (analfabeto, até oito anos de estudo, mais de oito anos de estudo); situação conjugal (com companheiro, sem companheiro); deficiência/transtorno (sim, não); local de ocorrência da violência (residência, via pública, outro); período do dia em que ocorreu a violência (manhã, tarde, noite, madrugada); se a violência ocorreu outras vezes (sim, não); vínculo agressor/vítima (parceiro, familiar, conhecido, desconhecido, a própria pessoa, outros); tipo de violência (autoprovocada, física, psicológica, física e psicológica, sexual, tortura, financeira, outros, ignorado); e meios de agressão (força física, ameaça, ameaça e força física, arma de fogo, objeto perfurocortante, enforcamento, objeto contundente, outros, ignorado). Por ser possível registrar mais de um tipo de violência e meio de agressão para cada notificação, essas variáveis foram classificadas de modo a se manterem em categorias mutuamente excludentes (8).

O tempo entre a primeira notificação de violência e o óbito foi classificado em até seis meses ou mais de seis meses, utilizando-se a diferença entre a data da primeira notificação no Sinan e a data de óbito do SIM. Para o pareamento, foram consideradas as notificações de violência e os registros de mortalidade, além das variáveis: nome, nome da mãe, data de nascimento e município de residência.

### Controle de viés

A classificação da mortalidade em causas violentas e não violentas seguiu o preconizado pela CID-10. A fim de minimizar o potencial viés de classificação que pode ocorrer nos registros de mortalidade de populações vulneráveis, as causas indeterminadas foram incluídas nas causas violentas, pois a literatura indica que óbitos classificados como causas indeterminadas migram para suicídio ou homicídio após análise por pareamento (9) e investigação do óbito (10).

### Tamanho do estudo

Das 3.034.939 notificações de violência registradas no Sinan no período 2014-2022, foram incluídas 37.533 relativas a vítimas autodeclaradas como pessoas trans de acordo com os critérios de elegibilidade. Após a reestruturação da base Sinan no formato longitudinal, foram considerados, para o pareamento e a análise de dados, 36.452 casos de violência interpessoal ou autoprovocada. O pareamento com o SIM recuperou 1.325 óbitos por causas violentas e não violentas entre os 16.606.876 óbitos no período 2014-2022.

### Métodos estatísticos

As frequências simples e relativas das covariáveis foram comparadas segundo o óbito por causas violentas com o teste qui-quadrado e o nível de significância de 5%. As causas de óbito, segundo os capítulos da CID-10, foram analisadas por meio de frequência relativa. As associações entre as covariáveis e o óbito foram medidas pela razão de prevalência (RP) com os respectivos intervalos de confiança de 95% (IC95%) por meio de regressão de Poisson. A qualidade do ajuste foi avaliada pelo pseudo R^2^, desvio da verossimilhança e critério de informação de Akaike corrigido. Todas essas análises foram conduzidas com o pacote blorr (11) no programa R.

### Acesso aos dados e métodos de limpeza

As notificações de violência interpessoal e autoprovocada e os registros de óbitos com identificação de nomes foram obtidos por meio de solicitação à Secretaria de Vigilância em Saúde e Ambiente, com assinatura dos autores do estudo do registro de responsabilidade e confidencialidade diante da cessão das bases de dados. A base de dados do Sinan é periodicamente submetida à análise de consistência e de dados faltantes, assim como a base do SIM.

### Pareamento dos dados

O Sinan admite mais de uma notificação para o mesmo indivíduo, razão pela qual as notificações foram transpostas em formato longitudinal, permitindo a observação do indivíduo ao longo do tempo, ou seja, a base de dados deixou de ser composta por notificações para ser formada por registros individuais. Para a transposição, foram usadas as variáveis consideradas em pareamento de dados: nome, data de nascimento, nome da mãe e município de residência.

Após a transposição longitudinal, a vinculação entre o SIM e o Sinan utilizou os campos de variáveis supracitadas e foi conduzida em quatro etapas.

Na primeira, na base longitudinal do Sinan e do SIM, os nomes do indivíduo e da mãe foram padronizados, excluindo-se caracteres especiais, espaços duplos, acentos, conectores, preposições e espaços em branco, bem como os campos relativos à data de nascimento. Um indexador único de sequência numérica foi criado nessas bases, e elas foram reduzidas em uma cópia, somente com os campos necessários ao pareamento.

Nas bases reduzidas, a segunda etapa foi a separação dos nomes em primeiro nome, último nome e nome do meio tanto da vítima quanto da mãe da vítima. Das duas bases reduzidas, os registros sem o nome do indivíduo foram excluídos.

A terceira etapa foi a vinculação determinística entre o SIM e o Sinan reduzidos. Foi criada uma chave única composta dos campos completos: nome, data de nascimento, sexo, nome da mãe e município de residência. Essa chave foi usada no pareamento das bases, considerando 100% de similaridade, com uso da função left_join do software R, ou seja, as informações de óbitos mediante pareamento da chave única foram acrescentadas ao banco reduzido do Sinan.

A quarta etapa foi a vinculação dos registros (não pareados anteriormente) por pareamento probabilístico, utilizando: primeiro nome, nome do meio e último nome do indivíduo e da mãe, sexo, data de nascimento e município de residência. O pareamento probabilístico utilizou o pacote RecordLinkage (12) do R, que permitiu a transformação dos campos de nomes em código fonético, por meio do uso do SoundexBR (13).

No pareamento, pesos foram atribuídos aos potenciais pares, sendo possível estabelecer um ponto de corte para verificação da similaridade dos registros. Entretanto, a população sob estudo foi específica, e optou-se pelo não estabelecimento de um ponto de corte. O pacote RecordLinkage permitiu a exportação dos potenciais pares em arquivo de formato CSV. A partir desse arquivo, os registros foram inspecionados manualmente, a fim de assegurar a identificação e a validação dos pares verdadeiros. 

Após a quarta etapa, os pares identificados no pareamento probabilístico foram integrados nas bases reduzidas por meio dos indexadores únicos, que já continha os pares identificados deterministicamente. Por fim, por meio da função left_join, essa base foi integrada à maior base do Sinan.

## Resultados 

Entre 2014 e 2022, foram notificados 36.452 casos de violência interpessoal ou autoprovocada em pessoas trans; 1.325 foram pareados a óbitos (3,6% do total das notificações) e, destes, 563 (42,5%) foram por violência (homicídio ou suicídio). Observou-se aumento das notificações ao longo dos anos, de 1,3% em 2014 para 16,2% em 2022 (Tabela 1).

**Tabela 1:** Frequência e proporção de notificações de violência e óbitos de pessoas trans, recuperadas pelos processos de pareamento determinístico e probabilístico, segundo ano da notificação e por causas violentas e não violentas. Brasil, 2014-2022 (n=36.452)

Ano	Notificações	Óbitos		
		Total	Causas violentas	Demais causas
	n (%)	n (%)	n (%)	n (%)
2014	491 (1,3)	20 (4,1)	19 (95,0)	1 (5,0)
2015	3.137 (8,6)	63 (2,0)	47 (74,6)	16 (25,4)
2016	4.285 (11,8)	99 (2,3)	54 (54,5)	45 (45,5)
2017	4.067 (11,2)	134 (3,3)	66 (49,3)	68 (50,7)
2018	4.654 (12,8)	135 (2,9)	52 (38,5)	83 (61,5)
2019	4.802 (13,2)	174 (3,6)	81 (46,6)	93 (53,4)
2020	4.269 (11,7)	205 (4,8)	66 (32,2)	139 (67,8)
2021	4.825 (13,2)	240 (5,0)	82 (34,2)	158 (65,8)
2022	5.922 (16,2)	255 (4,0)	96 (37,6)	159 (62,4)
Total	**36.452** (100,0)	**1.325** (3,6)	**563** (42,5)	**762** (57,5)

Agrupadas por capítulo da CID-10, causas externas corresponderam a 50,1% do total de óbitos; doenças do aparelho circulatório, a 10,9%; e doenças infecciosas e parasitárias, a 10,0% (Figura 1). Ao considerar as dez causas de óbitos mais frequentes entre todas, destacaram-se as agressões por arma de fogo (10,9%) e o suicídio por enforcamento, estrangulamento ou sufocação (10,4%). Os homicídios por arma de fogo e o suicídio por enforcamento, estrangulamento ou sufocação corresponderam a 50,0% do total de mortes violentas (Figura 1).

**Figura 1:** Proporção das principais causas de morte de pessoas trans que sofreram violência: (A) Proporção de óbitos por capítulos da décima revisão da Classificação Internacional de Doenças (CID-10); (B) causas da CID-10; (C) causas externas da CID-10. Brasil, 2014-2022 (n=1.325)

A análise das notificações registradas no Sinan das pessoas trans que vieram a óbito por causas violentas mostrou que a maioria dos casos notificados foi entre mulheres trans e travestis (73,5%), entre pessoas de 20 a 39 anos (54,2%), da raça/cor da pele parda (50,6%) e com até 8 anos de estudo (45,3%). Observou-se também que a maior parte dessas pessoas não possuía companheiro (67,6%) e não apresentava deficiência ou transtorno registrado na notificação (79,8%) (Tabela 2).

**Tabela 2:** Frequência e proporção das características das pessoas trans com notificação de violência, segundo óbito por causas violentas (n=563) e não violentas (n=762). Brasil, 2014-2022 (n=1.325)

Variáveis	Óbito por causas violentas		p-valor
	Sim^a^	Não^b^	
	n (%)	n (%)	
**Identidade de gênero**			0,006
Mulher trans e travestis	414 (73,5)	609 (79,9)	
Homem trans	149 (26,5)	153 (20,1)	
**Faixa etária** (anos)			<0,001
10-14	14 (2,5)	8 (1,0)	
15-19	88 (15,6)	34 (4,5)	
20-39	305 (54,2)	210 (27,6)	
40-59	115 (20,4)	183 (24,0)	
60+	41 (7,3)	327 (42,9)	
**Raça/cor da pele**			0,007
Branca	191 (35,4)	294 (41,2)	
Preta	55 (10,2)	75 (10,5)	
Amarela	3 (0,6)	6 (0,8)	
Parda	273 (50,6)	333 (46,6)	
Indígena	18 (3,3)	6 (0,8)	
Escolaridade			<0,001
Ensino fundamental	222 (45,3)	333 (49,0)	
Ensino médio	82 (16,7)	64 (9,4)	
Ensino superior	22 (4,5)	15 (2,2)	
Ignorado	164 (33,5)	268 (39,4)	
**Situação conjugal**			0,116
Com companheiro	165 (32,4)	241 (36,9)	
Sem companheiro	344 (67,6)	413 (63,1)	
**Deficiência ou transtorno**			0,458
Sim	102 (20,2)	154 (22,0)	
Não	403 (79,8)	547 (78)	
**Local de ocorrência**			<0,001
Residência	299 (55,8)	521 (71,6)	
Via pública	165 (30,8)	133 (18,3)	
Outros	72 (13,4)	74 (10,2)	
Turno			0,003
Manhã	75 (19,8)	90 (21,0)	
Tarde	76 (20,1)	129 (30,1)	
Noite	140 (37,0)	142 (33,1)	
Madrugada	87 (23,0)	68 (15,9)	
**Ocorreu outras vezes**?			<0,001
Sim	158 (34,3)	293 (45,5)	
Não	303 (65,7)	351 (54,5)	
**Lesão autoprovocada**			<0,001
Sim	243 (44,8)	148 (20,9)	
Não	299 (55,2)	561 (79,1)	
**Tipo de violência**			<0,001
Física	336 (61,4)	336 (45)	
Psicológica	8 (1,5)	19 (2,5)	
Física+psicológica	36 (6,6)	83 (11,1)	
Sexual	20 (3,7)	28 (3,8)	
Tortura	22 (4,0)	24 (3,2)	
Financeira	3 (0,5)	20 (2,7)	
Outras	122 (22,3)	236 (31,6)	
**Tipo de agressão**			<0,001
Força física	96 (17,6)	233 (34,1)	
Ameaça	3 (0,5)	15 (2,2)	
Ameaça+força física	10 (1,8)	31 (4,5)	
Arma de fogo	112 (20,5)	13 (1,9)	
Objeto perfurocortante	90 (16,5)	92 (13,5)	
Enforcamento	98 (17,9)	26 (3,8)	
Objeto contundente	23 (4,2)	37 (5,4)	
Outros	115 (21,0)	237 (34,6)	
**Vínculo agressor vítima**			<0,001
Parceiro	56 (11,2)	113 (16,2)	
Conhecido	64 (12,8)	69 (9,9)	
Familiar	22 (4,4)	179 (25,7)	
Desconhecido	116 (23,2)	105 (15,1)	
Própria pessoa	211 (42,1)	142 (20,4)	
Outros	32 (6,4)	88 (12,6)	
**Tempo entre notificação e óbito** (meses)			<0,001
Até 6	377 (67,0)	197 (25,9)	
Mais de 6	186 (33,0)	565 (74,1)	

^a^563 (42,5%); ^b^762 (57,5%).

Quanto às características das ocorrências notificadas, 55,8% dos casos aconteceram na residência da vítima, 37,0% ocorreram no período noturno e 65,7% tratavam-se de eventos registrados pela primeira vez, sem notificações anteriores. 44,8% dos casos foram de violência autoprovocada e 55,2% de violência interpessoasl. Entre a violência interpessoal, a violência física foi a mais frequente, representando 61,4% das notificações.

Os principais meios de agressão utilizados nesses episódios foram arma de fogo (20,5%), enforcamento (17,9%), força física (17,6%) e objetos perfurocortantes (16,5%). Quanto ao vínculo do agressor, 23,2% dos casos foram atribuídos a desconhecidos, 12,8% a conhecidos e 11,2% a parceiros íntimos.

Essas características referiram-se exclusivamente aos episódios de violência notificados previamente e não necessariamente à agressão que resultou no óbito.

Os resultados sobre os óbitos indicaram que determinadas características aumentaram a frequência de óbito por causas violentas entre as pessoas trans que haviam tido, em algum momento anterior, uma notificação de violência no Sinan.

Os resultados mostraram que a prevalência de óbito por violência foi maior entre pessoas trans mais jovens: 10-14 anos (RP 4,29; IC95% 2,82; 6,53), 15-19 anos (RP 3,82; IC95% 2,80; 5,21), 20-39 anos (RP 3,37; IC95% 2,52; 4,50) e 40-59 anos (RP 2,62; IC95% 1,94; 3,54), em comparação àquelas com 60 anos ou mais.

Entre as características das notificações anteriores, o risco de óbito violento foi maior nos indivíduos com registro de lesão autoprovocada (RP 1,50; IC95% 1,28; 1,76) e naqueles que sofreram agressões por arma de fogo (RP 2,00; IC95% 1,65; 2,43), enforcamento (RP 1,59; IC95% 1,36; 1,88), objeto contundente (RP 1,56; IC95% 1,10; 2,21) ou objeto perfurocortante (RP 1,48; IC95% 1,20; 1,83).

Além disso, pessoas cuja primeira notificação havia ocorrido há menos de 6 meses antes do óbito apresentaram maior prevalência de morte violenta (RP 2,07; IC95% 1,80; 2,39), o que indicou aumento da vulnerabilidade no período imediatamente posterior à notificação. 

## Discussão 

Os resultados indicaram aumento progressivo nas notificações de violência contra pessoas trans. Entre os casos registrados no Sinan que posteriormente evoluíram para óbito no SIM, observou-se maior frequência de mortes por causas violentas entre indivíduos na faixa etária de 10-14 anos, entre aqueles que apresentavam notificação de lesões autoprovocadas e que foram vítimas de agressões por arma de fogo, objetos perfurocortantes e enforcamento.

Tabela 3

**Tabela 3:** Razão de prevalência (RP) bruta e ajustada e intervalo de confiança de 95% (IC95%) de óbito por causas violentas em pessoas trans com notificação de violência, segundo variáveis do estudo. Brasil, 2014-2022 (n=1.325)

Variáveis	RP bruta (IC95%)	p-valor	RP ajustada (IC95%)	p-valor
**Identidade de gênero**				
Mulher trans e travestis	1,22 (1,06; 1,40)	0,038		
Homem trans	1,00			
**Faixa etária** (anos)				
10-14	5,71 (3,72; 8,76)	<0,001	4,29 (2,82; 6,53)	<0,001
15-19	6,47 (4,75; 8,82)	<0,001	3,82 (2,80; 5,21)	<0,001
20-39	5,31 (3,95; 7,16)	<0,001	3,37 (2,52; 4,50)	<0,001
40-59	3,46 (2,51; 4,78)	<0,001	2,62 (1,94; 3,54)	<0,001
60+	1,00		1,00	
**Situação conjugal**				
Com companheiro	1,00			
Sem companheiro	1,12 (0,97; 1,29)	0,238		
**Local de ocorrência**				
Residência	0,74 (0,61; 0,89)	0,021		
Via pública	1,12 (0,92; 1,36)	0,412		
Outros	1,00			
**Lesão autoprovocada**				
Sim	1,79 (1,58; 2,01)	<0,001	1,50 (1,28; 1,76)	<0,001
Não	1,00		1,00	
**Tipo de violência**				
Física	1,47 (1,25; 1,73)	<0,001		
Psicológica	0,87 (0,65; 1,21)	0,701		
Física+psicológica	0,89 (0,65; 1,21)	0,530		
Sexual	1,22 (0,85; 1,76)	0,404		
Tortura	1,40 (1,00; 1,96)	0,143		
Financeira	0,38 (0,13; 1,11)	0,100		
Outros	1,00			
**Meio de agressão**				
Força física	0,89 (0,71; 1,12)	0,413	1,28 (1,01; 1,62)	0,133
Ameaça	0,51 (0,18; 1,45)	0,249	0,67 (0,24; 1,87)	0,573
Ameaça+força física	0,75 (0,43; 1,31)	0,375	1,23 (0,74; 2,06)	0,541
Arma de fogo	2,74 (2,33; 3,22)	<0,001	2,00 (1,65; 2,43)	<0,001
Objeto perfurocortante	1,51 (1,23; 1,87)	0,003	1,48 (1,20; 1,83)	0,009
Enforcamento	2,42 (2,03; 2,88)	<0,001	1,59 (1,36; 1,88)	<0,001
Objeto contundente	1,17 (0,82; 1,67)	0,484	1,56 (1,10; 2,21)	0,077
Outros	1,00		1,00	
**Vínculo agressor vítima**				
Parceiro	1,24 (0,86; 1,79)	0,326		
Conhecido	1,80 (1,28; 2,55)	0,006		
Familiar	0,41 (0,25; 0,67)	0,001		
Desconhecido	1,97 (1,43; 2,72)	<0,001		
Própria pessoa	2,24 (1,64; 3,05)	<0,001		
Outros	1,00			
**Tempo entre notificação e óbito (meses)**				
Até 6	2,65 (2,31; 3,04)	<0,001	2,07 (1,80; 2,39)	<0,001
Mais de 6	1,00		1,00	

Entre as limitações do estudo, destacaram-se aquelas relacionadas ao uso de dados secundários, como a subnotificação dos casos, a incompletude e a inconsistência dos dados, o que pode comprometer a qualidade das análises e ocasionar vieses. 

Os achados deste estudo se limitaram à mortalidade de pessoas trans com notificação de violência no Brasil, portanto não revelaram a real violência sofrida pelas pessoas trans. Esta foi a primeira pesquisa que utilizou dados do Sinan e do SIM para explorar a temática da violência contra pessoas trans no Brasil. O ineditismo destes achados elucidou não apenas os fatores associados à mortalidade de pessoas trans, mas também a necessidade de mais estudos acerca da temática, em especial sobre gênero, expectativa de vida e a relação entre suicídio, gênero e faixa etária.

A inclusão do campo identidade de gênero na ficha de notificação de violência entre 2014 e 2015 resultou no aumento nos registros do Sinan ao longo dos anos. O resultado apresentado sugeriu que esse aumento pode ser decorrente das ações de treinamento para os profissionais que notificam esses eventos, o que resulta em maior sensibilidade na identificação e na notificação das violências que ocorrem nessa população, além de melhor preenchimento do campo. A ampliação da cobertura de municípios notificantes também pode ter contribuído para o aumento no número de casos de violência contra homens e mulheres trans e travestis. 

Embora este estudo não tenha identificado associação estatística entre identidade de gênero e óbito por causa violenta, a maioria dos casos ocorreu entre mulheres trans e travestis. Os homicídios ocorrem com maior frequência entre mulheres trans em comparação aos homens trans (6,14,15). Essa discrepância revelou a interseção de violências como transfobia, misoginia e racismo, que ampliaram sua vulnerabilidade (6,14,16). 

A prevalência de óbito por causas violentas foi maior em todas as faixas etárias quando comparada à de pessoas com 60 anos ou mais, diminuindo ao longo da vida. A expectativa de vida de pessoas trans foi considerada, em média, sete anos menor que a de pessoas não trans (18). Sendo assim, a maior ocorrência de óbito por homicídio ou por suicídio entre jovens pode indicar vulnerabilidades relacionadas a falta de apoio, discriminação, insegurança financeira, insatisfação corporal e questões emocionais (17). 

A violência física esteve presente na maior parte das notificações referentes a pessoas trans que posteriormente evoluíram para óbito. Após as agressões verbais, como ofensas e xingamentos, a violência física constituiu o principal tipo de agravo registrado, correspondendo a 62% dos casos (19).

As causas de óbito mais frequentes entre as pessoas trans foram agressão por disparo de arma de fogo, suicídio por enforcamento e agressões por objetos perfurocortantes. A análise dos fatores associados indicou que as mortes violentas foram mais recorrentes entre aquelas que apresentavam, nas notificações, registros de enforcamento, agressões por objetos contundentes e perfurocortantes e, especialmente, agressões envolvendo armas de fogo. Esse padrão contrastou com o da população em geral, cujas principais causas de morte foram relacionadas a doenças cardiovasculares e neoplasias (20).

Os indivíduos trans, especialmente aqueles com até 30 anos, foram os principais alvos de assassinatos nessa comunidade, além de sofrerem mais ferimentos do que o necessário para ocasionar em morte, frequentemente perpetrados por meio de armas brancas, espancamentos, asfixia e armas de fogo (21). Vítimas de agressões por esses meios devem ser cuidadosamente observadas pelos profissionais responsáveis pelo atendimento e pelo encaminhamento na rede de proteção, pois o uso desses métodos pode indicar maior agressividade por parte do agressor, o que aumenta o risco de reincidência da violência e de óbito subsequente (8).

A posse de armas de fogo esteve associada ao aumento da violência letal no país (22). A Lei nº 13.880/2019, ao flexibilizar a posse, contribuiu para o crescimento de registros de colecionadores, atiradores e caçadores. Por isso, é fundamental implementar políticas que restrinjam o acesso a armamentos como estratégia de enfrentamento da violência letal.

Em outro estudo, evidenciou-se que 29% das pessoas trans já haviam tentado suicídio ao longo da vida, o que é maior quando comparado aos estudos realizados na população geral (23). No Brasil, entre 278 pessoas trans, essa prevalência foi 43,1% entre os participantes. Destacou-se a alta incidência de transtornos mentais nessa população, especialmente a depressão (25).

A transfobia impacta negativamente a saúde física e mental dos indivíduos. Tal realidade pode se manifestar por meio de violência institucional, além de conflitos familiares, frequentemente resultando no afastamento do ambiente de convívio e na perda de moradia ou de abrigo (26). Ao longo da vida, a exposição a violências devido ao preconceito, à discriminação e à estigmatização pode impactar negativamente a saúde mental das pessoas vitimizadas, contribuindo para o maior risco ao comportamento suicida e às violências interpessoais (27,28). 

Este estudo identificou maior prevalência de óbitos por causas violentas nos seis meses após a primeira notificação e destacou a necessidade de respostas rápidas para proteger a integridade física dos agredidos, prevenir o agravamento da violência e os óbitos, além de garantir a segurança de pessoas trans em situação de violência.

Apesar da ausência de dados oficiais que permitam dimensionar com precisão a população trans no país, os achados deste estudo evidenciaram a gravidade da mortalidade por causas violentas nesse grupo, bem como a fragilidade dos sistemas de informação em captar, qualificar e integrar dados essenciais para a vigilância em saúde dessa população. Os achados reforçam a urgência de aprimorar os sistemas de informação, para permitir maior compreensão dos contextos de violência que afetam a população trans. Além disso, evidenciam a necessidade de políticas públicas inclusivas (especialmente voltadas aos indivíduos mais jovens) que promovam visibilidade, proteção e garantia de direitos, com foco na ampliação da expectativa e da qualidade de vida das pessoas trans. 

A elevada prevalência da mortalidade por causas violentas entre indivíduos com registros de violência autoprovocada, aliada ao curto intervalo entre a notificação e o óbito, evidencia a imprescindibilidade de vigilância ativa para a intervenção oportuna. É fundamental que os serviços de saúde estejam preparados para oferecer acolhimento qualificado e assistência integral, garantindo respostas oportunas e efetivas aos casos identificados. Sendo assim, é essencial que os profissionais da vigilância e da atenção estejam capacitados para reconhecer situações de maior vulnerabilidade e realizar os encaminhamentos adequados para os serviços especializados e para a rede de proteção.

## Data Availability

Por se tratarem de dados sensíveis e identificáveis, os bancos de dados utilizados nesta pesquisa não foram disponibilizados em repositórios públicos.
